# Staphylococcus pseudintermedius Bacteremia in a Lung Transplant Recipient Exposed to Domestic Pets

**DOI:** 10.7759/cureus.14895

**Published:** 2021-05-07

**Authors:** Coulter Small, Norman Beatty, Guy El Helou

**Affiliations:** 1 Division of Infectious Diseases and Global Medicine, Department of Medicine, University of Florida College of Medicine, Gainesville, USA

**Keywords:** bacteremia, staphylococcus pseudintermedius, lung transplant, multi-drug resistant bacteria

## Abstract

*Staphylococcus pseudintermedius *commonly colonizes companion animals, including canines. This microbe is a major opportunistic pathogen responsible for pyogenic and necrotizing skin and soft tissue infection in canines. Infection with *S. pseudintermedius* is increasingly being recognized in humans, especially in those who are immunocompromised*. *This microbe is quite similar to *Staphylococcus aureus*, expressing several analogous virulence factors and a variety of toxins. Furthermore, *S. pseudintermedius* has variants that display multi-drug resistance comparable to methicillin-resistant *S. aureus*. We report a 50-year-old female with bilateral lung transplant on immunosuppression who presents with signs of sepsis and pneumonia. Initial blood cultures grew Gram-positive cocci that were not initially identified via molecular diagnostics as *Staphylococcus *species but were later confirmed as *S. pseudintermedius* through mass spectrometry. Antimicrobial susceptibility testing demonstrated multi-drug resistance, including methicillin. Despite aggressive medical and antimicrobial treatment, our patients succumbed to the infection. The source of infection likely came from her companion canine at home as no other source could be identified; however, cultures were unable to be obtained from the companion canine. Those who are immunosuppressed, such as with solid organ transplants, should take caution with exposure to companion animals due to the potential for *S. pseudintermedius* infection.

## Introduction

*Staphylococcus pseudintermedius* is a common opportunistic pathogen in domestic canines and felines. This pathogen is known to cause skin/soft tissue and post-operative wound infections [1]. Human infection with *S. pseudintermedius* is increasingly being demonstrated and is now considered an emerging pathogen; displaying the ability to colonize human skin and mucous membranes [1]. Much of the current literature on the pathogenicity of *S. pseudintermedius* is in animals, with few case reports of known human infection [1,2]. *S. pseudintermedius* displays many similar features to *Staphylococcus aureus* and can commonly be confused on Gram staining and initial biochemical testing: both being Gram-positive cocci, coagulase-positive, and catalase-positive. *S. pseudintermedius* is capable of forming multiple layered biofilms, and variants display multi-drug resistance [1-5]. This combination can make *S. pseudintermedius* immensely pathogenic. With the known prevalence of multi-drug resistance in *S. pseudintermedius*, immunocompromised patients, such as those receiving solid organ transplantation, may need special education on the possibility of infection when living with common domestic animals.

## Case presentation

A 50-year-old female presented to our institution for the gradual onset of nonproductive cough, chills, and shortness of breath exacerbated by exertion. She reported that the symptoms started around 10 days prior to her presentation. She denied chest pain, fever, and rash.

Her past medical history was notable for bilateral lung transplant five years prior for end-stage lung disease due to progressive bronchiectasis, now on home oxygen 3 L/min via nasal cannula due to chronic lung allograft dysfunction (CLAD) of transplanted lungs with bronchiolitis obliterans syndrome, hypogammaglobulinemia, common variable immunodeficiency, previous left wrist fracture status post-open-reduction and internal fixation (ORIF) with a metal rod and screw placement four years earlier, and type II diabetes mellitus. Her current immunosuppressive regimen included cyclosporine 150 mg orally twice daily, mycophenolate mofetil 750 mg orally twice daily, and prednisone 5 mg daily. She was taking trimethoprim-sulfamethoxazole and valganciclovir for pneumocystis and cytomegalovirus prophylaxis, respectively. The patient reported living with two other family members, two dogs, and a cat. Denies any history of travel, intravenous drug use, or high-risk activities. Prior to her presentation, the patient started a course of levofloxacin prescribed by her primary care provider, with no improvement, and ultimately presented to the emergency department.

At presentation, the patient had tachycardia to 97 bpm, a BP of 117/70 mmHg, a temperature of 36.9 °C (98.4 °F), a respiration rate of 21 breaths per minute, and a SpO_2_ of 90-95% on 5 L nasal cannula. The physical examination was notable for shortness of breath with exertion, decreased breath sounds with bilateral pulmonary rales, and mild swelling and tenderness to palpation of left wrist overlaying her previous surgical site. Her admission laboratory results are summarized in Table 1.

**Table 1:** Initial blood test results. BUN: blood urea nitrogen, AST: aspartate aminotransferase, ALT: alanine aminotransferase.

Marker	Reference value	Patient’s value
WBC	4.0–10 thou/cm^3^	7.7 thou/cm^3^
Neutrophils	40.0–80.0%	79%
Lymphocytes	20.0–45.0%	12.5%
Monocytes	2.0–10.0%	5.9%
Eosinophils	0.0–8.0%	1.7%
Basophils	0.0–2.0%	0.9%
Platelet count	150–450 thou/cm^3^	210 thou/cm^3^
Hemoglobin	12.0–16.0 g/dL	9.8 g/dL
Hematocrit	35.0–45.0%	31.8%
C-reactive protein	0.00–5.00 mg/L	234.41 mg/L
Albumin	3.5–5.2 g/dL	3.0 g/dL
Creatinine	0.38–1.02 mg/dL	0.76 mg/dL
BUN	6–21 mg/dL	11 mg/dL
Lactic acid	0.3–1.5 mmol/L	0.7 mmol/L
AST	0–37 IU/L	15 IU/L
ALT	0–35 IU/L	6 IU/L
Total bilirubin	0.0–1.0 mg/dL	0.4 mg/dL
Direct bilirubin	0.0–0.2 mg/dL	<0.1 mg/dL
Alkaline phosphatase	33–133 IU/L	148 IU/L

**Figure 1:**
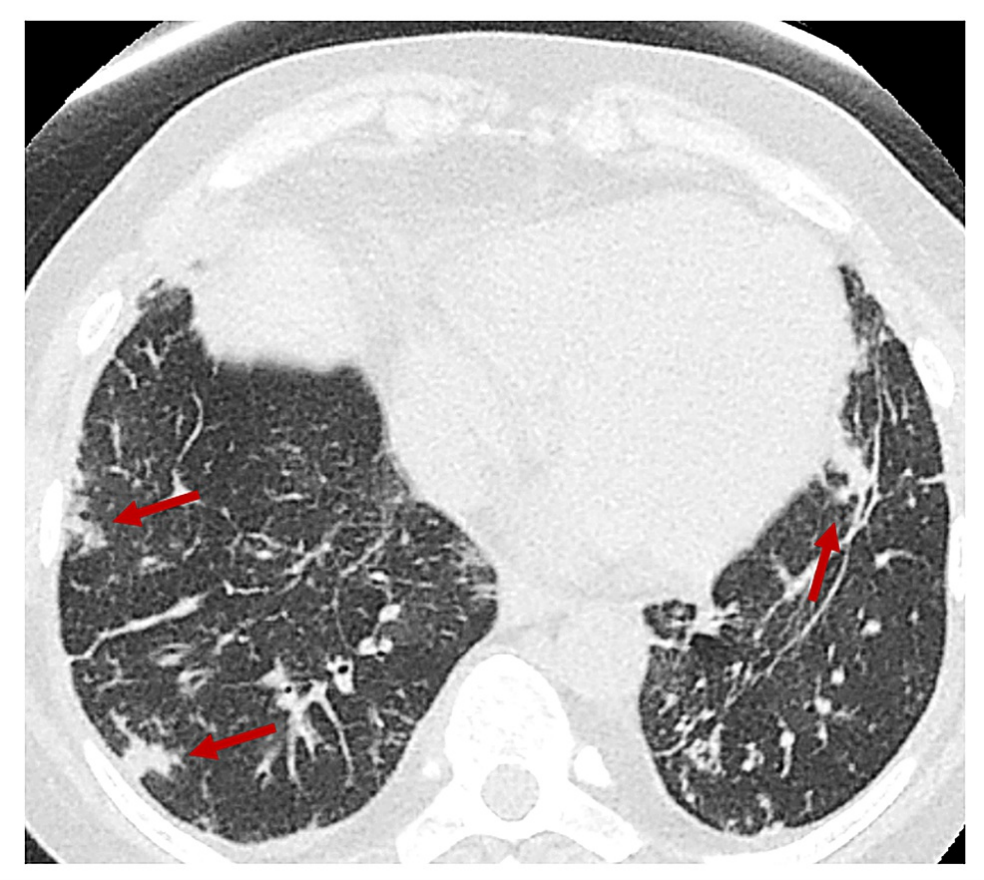
Chest CT without contrast at admission showing multiple areas of nodularity (red arrows) revealing possible septic embolic disease.

Due to the COVID-19 pandemic and her acute presentation of shortness of breath, COVID-19 was ruled out by a nasopharyngeal swab polymerase chain reaction (PCR). Chest X-ray on admission was concerning for right lower lobe pneumonia. Initially, she responded well to supplemental oxygen, bronchodilator therapy, and empiric antibiotics, which were cefepime and vancomycin. However, over the course of the next few days, her respiratory status worsened with increasing hypoxia, and she was transferred to the medical intensive care unit (MICU). A CT of the chest was obtained and was notable for diffuse and multifocal tree-in-bud opacities, concerning the ongoing infection (Figure 1).

**Figure 2:**
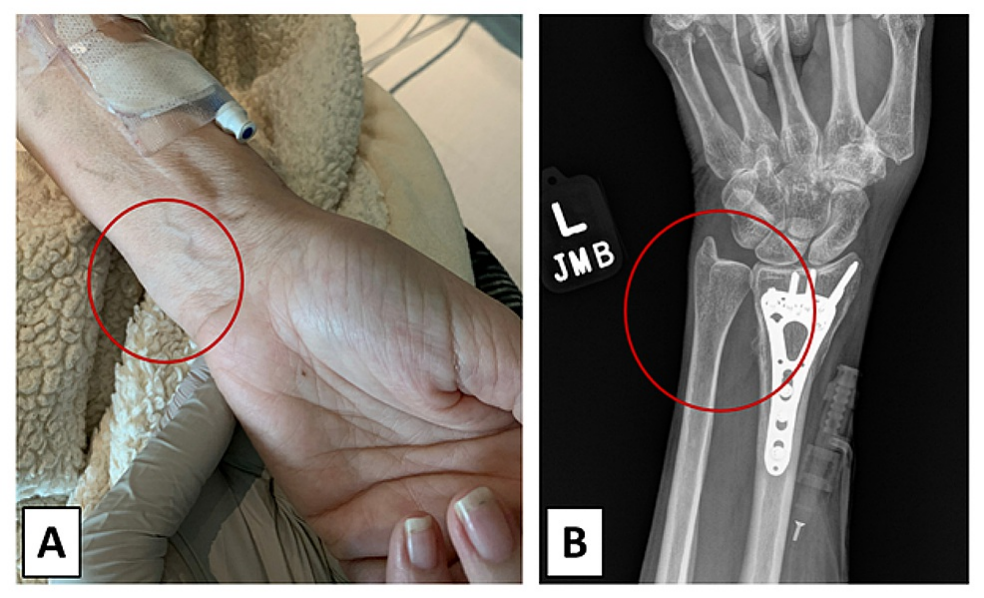
Investigation of the left wrist for infection on hospital day 8. (A) Gross observation of left wrist and (B) X-ray of patient's left wrist.

Blood cultures from admission grew at 21 hours from the two anaerobic bottles of Gram-positive cocci that were not identified by The BioFire® FilmArray® Blood Culture Identification Panel (BioFire, Salt Lake City, UT). The organisms were plated on sheep-blood agar where colonies had a white-gray appearance and exhibited β-hemolysis. Furthermore, the microorganism in question was both catalase and coagulase-positive via biochemical testing. It was subsequently identified by the VITEK® SOLUTIONS automated mass spectrometry (Vitek Solutions, Inc., Fenton, MO) microbial identification system as *S. pseudintermedius*. The in vitro minimal inhibitory concentrations can be found in Table 2. Follow-up blood cultures, obtained after empiric antibiotics were all negative. Antibiotics were narrowed to intravenous vancomycin with a goal trough of 15 to 20 µg/mL, and cefepime was stopped. Transthoracic and transesophageal echocardiogram failed to identify valvular vegetations. The wrist was suspected as the source due to the patient’s complaint of tenderness to palpation and movement overlaying the hardware, and an X-ray was obtained (Figure 2), with no abnormalities except swelling abutting orthopedic hardware.

**Table 2:** Staphylococcus pseudintermedius in vitro susceptibilities.

Antibiotic	Minimum inhibitory concentration	Susceptibility
Ciprofloxacin	≥8	Resistant
Daptomycin	0.25	Susceptible
Erythromycin	≥8	Resistant
Gentamicin	≥16	Resistant
Levofloxacin	≥8	Resistant
Linezolid	2	Susceptible
Minocycline	4	Susceptible
Moxifloxacin	4	Resistant
Oxacillin	≥4 µg/mL	Resistant
Penicillin	≥0.5 µg/mL	Resistant
Rifampin	≤0.5	Susceptible
Tetracycline	≥16	Resistant
Trimethoprim-sulfamethoxazole	≥320	Resistant
Vancomycin	1	Susceptible

Left wrist swelling (Figure 2A; red circle) was noted on physical examination; furthermore, the left wrist was tender to palpation and can be seen on X-ray (Figure 2B; red circle) abutting orthopedic hardware in distal radius (Figure 2).

An orthopedics consultation was requested and noted a palpable fluid collection over the flexor tendons. Continued medical management was recommended, given clinical improvement, and awaiting MRI results. MRI of the left wrist was attempted, but the patient could not tolerate the procedure, due to anxiety.

**Figure 3:**
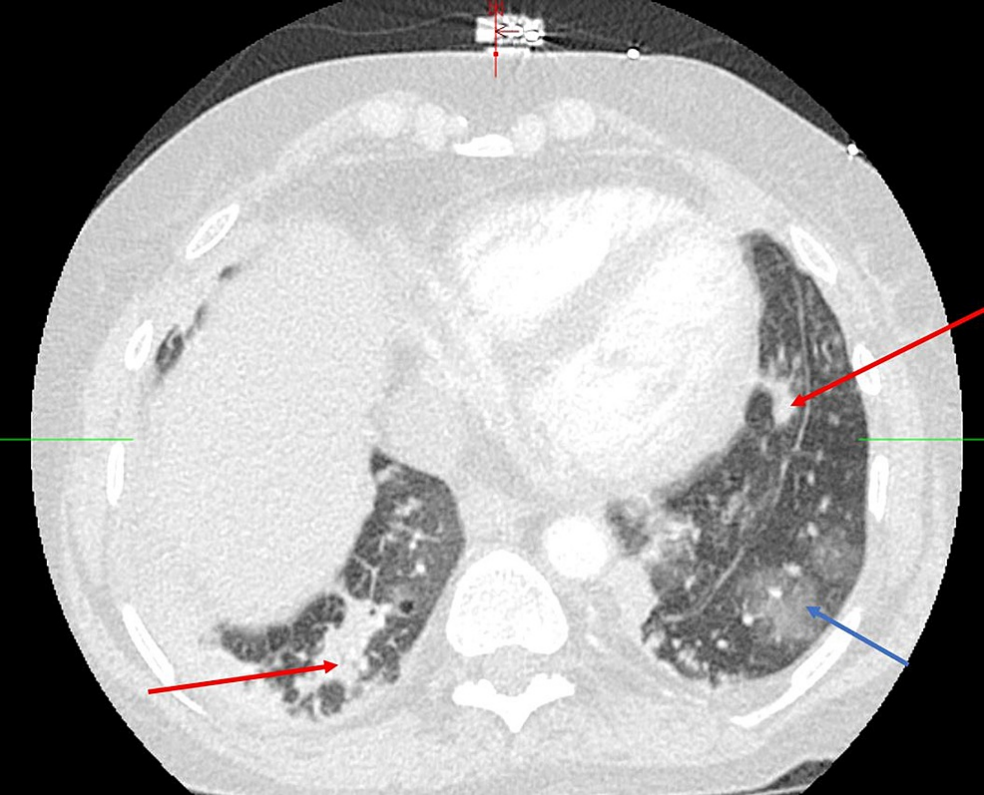
Computed tomography angiography chest on hospital day 9.

On hospital day nine, the patient developed hypoxic respiratory distress with sepsis evidenced by tachycardia, tachypnea, fever of 38.5 °C (101.3 °F), and increasing leukocytosis. Her antibiotics were broadened again with the addition of cefepime. Blood and sputum cultures were obtained and had no growth (blood) and normal flora (sputum). A CT angiography was obtained, and multifocal opacities in both lung bases with ground-glass opacities in the lingula were noted (Figure 3). At that point, it was not clear if these opacities represented continued infection versus worsening of her underlying CLAD.

Bilateral consolidations (red arrows), as well as ground-glass opacities (blue arrow) in the lingula, were noted in Figure 3. At the wishes of the patient and due to her chronic illnesses (CLAD with no plans of re-transplantation), she was placed on comfort care and passed away three days later. Unfortunately, no definitive vector for *S. pseudintermedius *infection was determined, as her companion canine was unable to be tested for colonization.

## Discussion

Colonization with *S. pseudintermedius* is common in pet canines; Hansleman et al. described 61 of 161 (46%) dogs in their cohort to be colonized with *S. pseudintermedius* [6]. This proportion was as high as 76.8% and 87.4% with other groups [7,8]. Adding to the complexity is the prevalence of drug resistance in *S. pseudointermedius* [7-11]. Videla et al. reported 71.1% of isolates recovered from the USA demonstrated resistance to at least three antimicrobial classes, other than beta-lactams [9]. Ruzauskas et al. described resistance patterns ranging from 31% to ciprofloxacin, 65% to tetracyclines, 69% to macrolides, and 94% to penicillin G [7]. Several virulence factors have been implicated in disease severity in both human and dogs, including leukocyte specific leukotoxin Luk-I and phenol-soluble modulins (PSMs), which can disrupt cell membranes of a variety of cell types [12]. Methicillin-resistant* Staphylococcus pseudointermedius* (MRSP) has emerged at a global level and has been described in the canine population. Clones harboring specific fluoroquinolones resistance genes GyrA+GrIA seem to also harbor other prophages carrying genes associated with increased virulence and fitness [13]. Fortunately, these clones also exhibit genetic insertion within the comGA gene, which plays a major role in horizontal gene transfers, hence making them less likely to share their genetic material.

Though human infection is believed to be rare, because the organism is prevalent in canines and has the potential to harbor a variety of virulence factors and drug-resistance genes, it has the potential to cause devastating infections in the immunocompromised host. Our patient’s bacteremia cleared quickly with the initiation of vancomycin; however, that may not be the rule in this patient population. Her worsening clinical status and respiratory distress were believed to be due to her low reserve lung function and CLAD, and further invasive investigations such as bronchoscopy were not done respecting the patient’s wishes for comfort care. Could a cytokine storm effect caused by bacteriolysis and release of preformed Luk-I and PSM toxins explain her rapid deterioration following appropriate antibiotic therapy? With her negative blood and sputum cultures, worsening respiratory status, and bilateral consolidations, and ground-glass opacities, we believe this could be a possibility. Though the patient denies any wounds caused by bites or scratches from her dogs, she was likely exposed by her pet canine, which may have been colonized by *S. Pseudintermedius*. Though post-transplant education includes advice about pet handling and care, it may be useful to emphasize handwashing after contact with home pets.

Another potential concern with* S. pseudointermedius* is the inability of a rapid blood culture identification technique to identify it as seen in our patient with The BioFire® FilmArray® Blood Culture Identification Panel (BioFire Diagnostics, Salt Lake City, UT). As these panels typically identify all major pathogenic Gram-positive cocci, this may lead to wrong assumptions of blood culture contaminants, especially when bacteremia is not persistent.

Previous reports of *S. pseudointermedius* infections in humans have been published [2,4,14-16]. In a report on 24 human infections, 18 (75.0%) were soft tissue infections, and two (8.3%) were invasive: a bloodstream infection and a prosthetic joint infection [2]. The *S. pseudointermedius* cultured from both the bloodstream infection and prosthetic joint infection demonstrated resistance to ampicillin and penicillin and were treated with vancomycin and tigecycline, respectively [2]. From the data available, 95.4% of cases reported having a pet dog [2]. A separate experience documented four cases of infection: skin lesions in three and sepsis in one patient [14]. All four had documented MSRP infection [14]. The patient who presented with sepsis had a past medical history relevant for a liver transplant and was immunosuppressed; however, other bacteria were also identified in their blood cultures, including *Klebsiella pneumoniae*, *Citrobacter spp.*, and *Enterococcus faecium* [14]. Another case report describes an MRSP infection complicating a skin Graft-versus-Host disease in a bone marrow transplant recipient [16]. This particular isolate demonstrated resistance to benzyl-penicillin, oxacillin, fluoroquinolones, macrolides, clindamycin, and trimethoprim-sulfamethoxazole [16]. This again highlights the opportunistic nature of *S. pseudointermedius* and its potential for multidrug resistance.

## Conclusions

Overall, *S. pseudintermedius* is an emerging human pathogen with the potential to cause life-threatening infections due to its virulence, fitness factors, drug-resistance genes, and stealth when it comes to common rapid detection panels. Provider awareness is important, and we believe that a Gram-positive cocci bacteremia, even in one bottle, in a critically ill patient should not be immediately dismissed if not identified by blood culture identification panels. This is even more important when the host is immunocompromised and has had contact with pets, notably dogs.
